# Impact of mirikizumab on patient-reported outcomes and quality of life in patients with Crohn’s disease: results from the phase 2 SERENITY study

**DOI:** 10.1093/crocol/otag018

**Published:** 2026-03-11

**Authors:** Vipul Jairath, Theresa Hunter Gibble, Laurent Peyrin-Biroulet, Bruce E Sands, Fumihito Hirai, Toshifumi Hibi, Edward V Loftus, Raymond K Cross, Marijana Protic, Lai Shan Chan, Nathan Morris, Kristina Traxler, David T Rubin

**Affiliations:** Department of Medicine, Division of Gastroenterology, Western University, London, ON, Canada; Immunology, Eli Lilly and Company, Indianapolis, IN, USA; Department of Gastroenterology, University Hospital of Nancy, Nancy, France; Division of Gastroenterology, Icahn School of Medicine at Mount Sinai, New York, NY, USA; Department of Gastroenterology, Fukuoka University, Fukuoka, Japan; Center for Advanced IBD Research and Treatment, Kitasato University Kitasato Institute Hospital, Tokyo, Japan; Division of Gastroenterology and Hepatology, Mayo Clinic College of Medicine and Science, Rochester, MN, USA; Inflammatory Bowel Disease Program, University of Maryland School of Medicine, Maryland, MD, USA; Immunology, Eli Lilly and Company, Indianapolis, IN, USA; Immunology, Eli Lilly and Company, Indianapolis, IN, USA; Immunology, Eli Lilly and Company, Indianapolis, IN, USA; Immunology, Eli Lilly and Company, Indianapolis, IN, USA; Inflammatory Bowel Disease Centre, University of Chicago Medicine, Chicago, IL, USA

**Keywords:** quality of life, Crohn’s disease, mirikizumab

## Abstract

**Background:**

Mirikizumab is an anti-IL23p19 antibody that has shown efficacy in treating moderately to severely active Crohn’s disease in a phase 2 study. We studied mirikizumab’s impact on quality of life in these patients.

**Methods:**

Patients (*N* = 191) were randomized using a 2:1:1:2 allocation across 4 treatment arms (placebo, 200 mg, 600 mg, or 1000 mg mirikizumab, administered intravenously every 4 weeks [week 0, week 4, and week 8]). Patients who received mirikizumab and achieved ≥1-point improvement in Simple Endoscopic Score for Crohn’s Disease at week 12 were rerandomized into double-blind maintenance to continue treatment with either intravenous assignment or 300 mg mirikizumab subcutaneous every 4 weeks to week 52. Non-improvers or placebo patients received 1000 mg mirikizumab until week 52. Patients with clinical benefit from the maintenance period received 300 mg subcutaneously to week 104. Quality of life and patient-reported outcomes were evaluated with the Inflammatory Bowel Disease Questionnaire, 36-Item Short-Form Health Survey Mental Component Summary and Physical Component Summary, Patient’s Global Rating of Severity, and Abdominal Pain Numeric Rating Scale. Quality of life measures were evaluated up to week 104 and analyzed with a mixed model for repeated measures up to week 12 and descriptively afterward.

**Results:**

At week 12, all mirikizumab groups had improved quality of life and patient-reported outcome scores compared to placebo. These improvements were sustained through week 52 and week 104.

**Conclusions:**

Treatment with mirikizumab was associated with significantly improved quality of life and patient-reported disease severity sustained through week 104 in patients with moderately-to-severely active Crohn’s disease in this phase 2 study (NCT02891226).

## Introduction

Crohn’s disease (CD) is a chronic inflammatory disease of the gastrointestinal tract that includes the typical symptoms of abdominal pain, weight loss, chronic diarrhea, and fatigue.^1^ Approximately 70% of patients will develop structuring or penetrating complications after 10 years of CD diagnosis, with up to 40% of patients developing fistulae.^2^^,^^3^ Collectively, the symptoms and complications of CD have a significant negative impact on patient’s QoL.^4^^,^^5^

Additionally, restoration of QoL and reduction in disability are considered important long-term treatment targets that should be assessed in patients with CD.^6^ QoL is a multidimensional construct that focuses on patients’ perceptions of physical, psychological, and social functions.^7^ Symptoms from CD can severely impact patients’ QoL, such as sleep quality, pain, fatigue, loss of social satisfaction, and relation to depression and anxiety.^8–13^ The 2024 European Crohn’s and Colitis Organization guidelines for CD include and emphasize the importance of improving patients’ QoL, including patient-centered outcomes like mental health, daily functionality, and long-term well-being.^14^ Patient perspectives on QoL and patient-reported outcomes are important and impactful on patients’ acceptance and adherence to therapy.^15^

Current pharmacologic treatment for moderate-to-severe CD includes corticosteroids, immunomodulators, and biologics that target different mechanisms of action^16^^,^^17^; however, inadequate primary response, secondary loss of response, and poor drug tolerance limit the efficacy of available treatments.^16–19^ Suboptimal disease control has been associated with impaired QoL, higher surgery rates, increased hospitalization rates, and prolonged corticosteroid use.^20^^,^^21^ The STRIDE-II initiative has also marked QoL restoration as a crucial long-term treatment goal, independent of the typical treatment targets such as achieving clinical remission, endoscopic healing, and the normalization of inflammatory biomarkers.^6^

Mirikizumab is a humanized immunoglobulin G4 (IgG4), a variant monoclonal antibody that binds to the p19 subunit of IL-23 and has demonstrated safety and efficacy in the treatment of ulcerative colitis (UC) and CD.^22^^,^^23^ Mirikizumab treatment improved QoL in UC as early as week 12 (W12) and was sustained through W52.^23^^,^^24^

We aimed to evaluate the effect of mirikizumab on patient-reported outcomes (PROs) and QoL in patients as part of SERENITY, a phase 2 study in adult patients with moderately-to-severely active CD.

## Materials and methods

### Study design and participants

**Figure 1:**
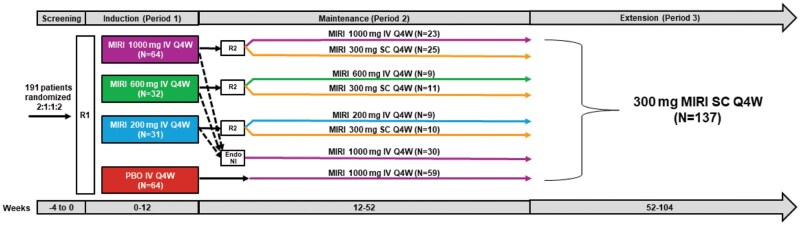
Study design for AMAG. Dashed lines indicate endoscopic non-improvers (NI) while solid lines indicate endoscopic improvers at the end of Period 1. R1 = Randomization 1: patients were stratified based on previous exposure to biologic therapy for the treatment of CD. R2 = randomization 2: patients who received mirikizumab during induction and had endoscopic improvement were re-randomized in a 1:1 ratio at week 12 to continue their induction regimen or receive mirikizumab 300 mg SC Q4W with stratification based on endoscopic response. All patients who received PBO in period 1 received mirikizumab 1000 mg IV Q4W. Abbreviations: CD = Crohn’s disease; IV = intravenous; MIRI = mirikizumab; PBO = placebo; Q4W = every 4 weeks; SC = subcutaneous.

SERENITY was a multicenter, randomized, parallel-arm, double-blind, placebo (PBO)-controlled study (Figure 1) conducted across 80 sites in 14 countries. Enrollment started in 2017, and the last patient visit was in 2019 (see Supplementary Appendix for CONSORT diagram).

Eligible patients were 18-75 years old who had a duration of active moderate-to-severe CD of ≥3 months, defined as stool frequency (SF) ≥4 and/or abdominal pain (AP) ≥2 at baseline and a centrally read Simple Endoscopic Score for Crohn’s Disease (SES-CD) ≥7 for patients with ileal-colonic or ≥4 for patients with isolated ileal disease within 14 days before the first dose of study treatment. Patients must have received prior therapy for CD, including a history of intolerance or inadequate response to aminosalicylates, 6-mercaptopurine, azathioprine, or corticosteroids or a history of corticosteroid dependence and/or have received treatment with ≥1 biologic agent (tumor necrosis factor antagonists, vedolizumab, investigational biologic CD therapeutics, etc.). Concomitant treatment with oral 5-aminosalicylic compounds, oral corticosteroids, azathioprine, 6-mercaptopurine, methotrexate, or CD-specific antibiotics was allowed.^22^

Patients were ineligible if they had the following: complications of CD, including strictures, stenoses, or any other manifestation for which surgery might be indicated or could confound the evaluation of efficacy; any bowel resection or diversion within 6 months or further intra-abdominal surgery within 3 months; the presence of stoma; previous exposure to any other biologic therapy targeting IL-23 p19 or ustekinumab (in a United States [US]-specific addendum, a single dose of ustekinumab was allowed if given at least 12 weeks before the baseline); received natalizumab or agents that deplete B or T cells within 12 months of screening; or been treated with any investigational drug for CD within 8 weeks before baseline or 5 half-lives of the drug (whichever is longer) or with interferon therapy within 8 weeks before baseline.

### Randomization and blinding

#### Induction

Patients were randomized in a 2:1:1:2 ratio across treatment groups, including PBO, 200 mg mirikizumab, 600 mg mirikizumab, or 1000 mg mirikizumab that was administered intravenously (IV) every 4 weeks (Q4W) through week 12.^22^ The randomization was stratified by prior exposure to biologic therapy for CD treatment. Prior biologic use was approximately 60.0% in all mirikizumab groups and 67.2% in the PBO group, with prior biologic failure rates of approximately 50.0% and 56.3% in the mirikizumab groups and PBO group, respectively (Table 1).

**Table 1:** Baseline demographics and clinical characteristics.

Mean (SD) unless otherwise specified	Treatment groups			
		Miri		
	Placebo (*N* = 64)	200 mg (*N* = 31)	600 mg (*N* = 32)	1000 mg (*N* = 64)
**Age, years**	39.0 (13.0)	38.1 (11.8)	40.4 (13.3)	37.7 (13.1)
**Male, *n* (%)**	28 (43.8)	17 (54.8)	14 (43.8)	34 (53.1)
**Race-White, *n* (%)**	55 (85.9)	28 (90.3)	24 (75.0)	52 (81.3)
**Disease duration, years**	10.2 (9.8)	8.9 (7.4)	10.8 (9.7)	8.6 (6.7)
**Disease location, *n* (%)**				
** Ileal**	11 (17.2)	6 (19.4)	5 (15.6)	11 (17.2)
** Colonic**	25 (39.1)	14 (45.2)	10 (31.3)	26 (40.6)
** Ileocolonic**	28 (43.8)	11 (35.5)	17 (53.1)	27 (42.2)
**Simple endoscopic score for Crohn’s disease (SES-CD)**	11.9 (5.6)	14.4 (7.9)	15.2 (7.4)	13.1 (6.8)
**PRO scores**				
**Stool frequency**	6.4 (3.1)	7.4 (3.0)	6.4 (3.8)	6.6 (5.5)
** Abdominal pain**	1.9 (0.6)	2.0 (0.6)	1.7 (0.7)	1.9 (0.6)
**Crohn’s disease activity index (CDAI)**	304.7 (93.1)	348.3 (92.1)	298.2 (103.7)	304.5 (94.4)
**Previous biologic use** ^a^ **, *n* (%)**	43 (67.2)	19 (61.3)	19 (59.4)	39 (60.9)
**Previous biologic failure** ^b^ **, *n* (%)**	36 (56.3)	15 (48.4)	16 (50.0)	31 (48.4)
**Prior vedolizumab use, *n* (%)**	14 (21.9)	5 (16.1)	5 (15.6)	6 (9.4)
**Prior anti-TNF use, *n* (%)**				
** 0**	25 (39.1)	14 (45.2)	14 (43.8)	26 (40.6)
** 1**	16 (25.0)	10 (32.3)	9 (28.1)	22 (34.4)
** 2**	22 (34.4)	7 (22.6)	5 (15.6)	14 (21.9)
** 3+**	1 (1.6)	0	4 (12.5)	2 (3.1)
**Concomitant oral corticosteroid use, *n* (%)**	21 (32.8)	14 (45.2)	7 (21.9)	15 (23.4)
**Concomitant immunosuppressant use, *n* (%)**	19 (29.7)	12 (38.7)	10 (31.3)	21 (32.8)
**IBDQ**	113.88 (37.07)	104.77 (34.31)	127.03 (35.47)	120.31 (32.40)
**hsCRP (median, Q1, Q3)**	6.8 (1.8, 19.0)	7.4 (2.3, 31.4)	6.8 (2.7, 20.7)	4.5 (2.7, 15.5)
**FCP (median, Q1, Q3)**	799.5 (256.5, 1945.5)	877.0 (225.0, 4359.0)	822.5 (355.0, 2302.5)	773.0 (293.0, 1634.0)

Intent-to-treat population.

a Although prior induction dosing of ustekinumab (UST) use was allowed, no patients had prior UST treatment.

b Inadequate response, loss of response, or intolerance to medication. Patients with prior biologic exposure that were not biologic failures discontinued treatment for the following reasons: cannot afford, treatment availability, subject decision, completed treatment, and other.

Abbreviations: FCP = fecal calprotectin; hsCRP = high-sensitivity C-reactive protein; IBDQ = Inflammatory Bowel Disease Questionnaire; Miri = mirikizumab; PRO = patient-reported outcomes; SD = standard deviation; TNF = tumor necrosis factor.

#### Maintenance

All patients received IV and subcutaneous (SC) dosing in a double-dummy design during the maintenance period (week 12–52) to maintain blinding.

##### Rerandomized maintenance cohort

All patients who received mirikizumab in the induction phase (week 0–12) and who also achieved an improvement (at least 1–point decrease) in their SES-CD score from baseline at W12 were then randomized evenly to either (1) continue induction treatment assignment (IV of 200, 600, or 1000 mg mirikizumab Q4W) or (2) receive IV PBO Q4W and SC 300 mg mirikizumab Q4W administered through W52. Randomization was stratified on the endoscopic response (achieved ≥1-point improvement at W12 in SES-CD).

##### Nonrandomized maintenance cohort

Patients who received mirikizumab during induction and did not achieve improvement from baseline SES-CD score at W12 (endoscopic non-improvers [NI]) and all patients who received PBO during induction received 1000 mg mirikizumab IV and SC PBO Q4W through W52.

A study site pharmacist or other trained person was unblinded at the site for investigational product preparation. Patients who met the enrollment criteria were randomized to the study drug at the baseline visit. Assignment to the double-blind investigational product was determined using a computer-generated random sequence using an interactive web-response system, and the site was responsible for administering the study drug to the patients.

#### Extension

All patients with clinical benefit per investigator continued open-label treatment from W52-W104 receiving 300 mg SC mirikizumab Q4W. Patients who did not have clinical benefit at W52 discontinued treatment and entered a follow-up period to assess their safety for an additional 16 weeks (W104-W120).

### Outcome measures

The main objective of this disclosure is to evaluate the treatment of mirikizumab across the treatment arms on PROs and QoL at W12, W52, and W104.

QoL endpoints included the following: change from baseline in the Inflammatory Bowel Disease Questionnaire (IBDQ) score (scores range from 32-224; a higher score indicates a better QoL) at W12, W52, and W104^25^; Medical Outcomes 36-item Short-Form Health Survey (SF-36) (a 36-item patient-completed measure designed to be a short, multipurpose assessment of health in the areas of physical functioning, role-physical, role-emotional, bodily pain, vitality, social functioning, mental health, and general health)^26^; IBDQ response was defined as ≥16-point improvement in IBDQ score, and IBDQ remission was defined as total IBDQ score ≥170.^27^^,^^28^

PROs included Abdominal Pain (AP) Numeric Rating Scale (NRS; measures “worst AP in the past 24 hours” using an 11-point scale [0 = no pain and 10 = worst possible pain] which patients recorded in an electronic diary tool); Patient’s Global Rating of Severity (PGRS) scale (1-item patient-rated questionnaire designed to assess the patients’ rating of their disease symptom severity over the past 24 hours).

### Statistical analysis

QoL/PRO outcomes at W12 were assessed in the intent-to-treat (ITT) population, which included all patients who were randomly assigned, and patients from the ITT population who entered the maintenance and extension periods were used to assess QoL/PRO outcomes at W52 and W104, respectively. Changes in QoL/PRO measures from baseline to W12 were compared between treatment groups using a mixed model for repeated measures (MMRM). This model includes treatment, geographic region, prior CD biologic therapy, visit, and visit-by-treatment interactions. QoL/PRO measures at W52 and W104 are presented descriptively due to low number of patients between the treatment arms and the lack of a placebo control group.

### Ethical considerations

The study complied with the International Conference on Harmonisation (ICH) guidelines on good clinical practice. Appropriate ethical review boards approved all informed consent forms and protocols before the initiation of the study. All patients provided written informed consent before receiving the study drug.

## Results

### Study population

In this study, 191 patients were randomized across the 4 treatment arms. Baseline characteristics were generally similar across the treatment groups. The average duration of CD was similar in all groups, including average duration of CD, AP, and SES-CD. Average SF and percentage of patients receiving oral corticosteroids or immunomodulators were numerically higher in the 200 mg group compared to the other groups (Table 1).

Of the 191 patients who began the induction period, 92.1% (176/191) went on to the maintenance period (W12-W52). Of those 176, 137 patients entered the extension period, and 124 patients completed treatment through W104 (Figure 1).

### QoL and PRO outcomes: induction period (W0-12)

**Figure 2:**
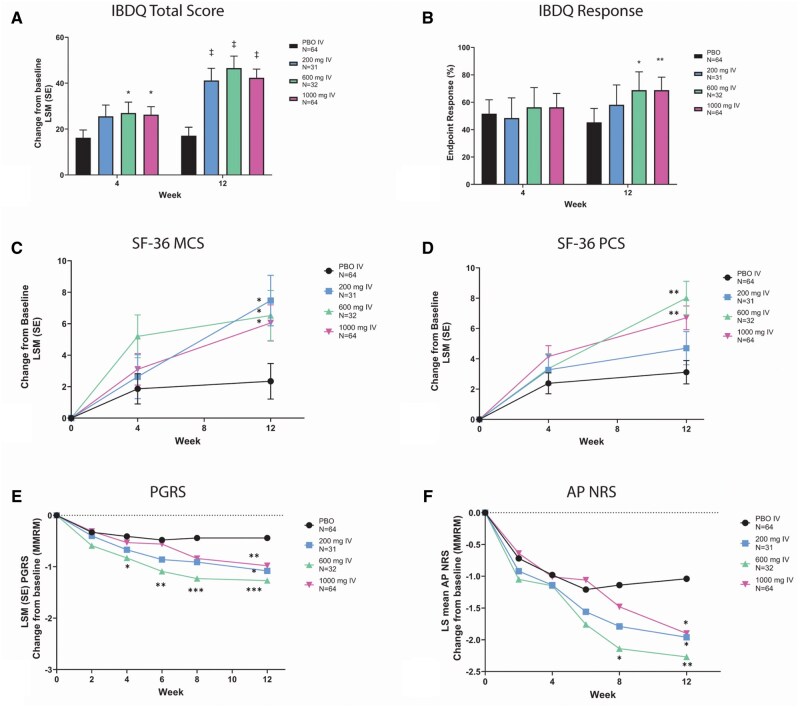
QoL and PRO assessments in the induction period (week 0-12) including change from baseline in IBDQ total score (A), IBDQ response (B), change from baseline in SF-36 MCS (C) and PCS (D), change from baseline in PGRS (E), and change from baseline in AP NRS (F). Abbreviations: AP NRS = Abdominal pain Numeric Rating Scale; IBDQ = Inflammatory Bowel Disease Questionnaire; LSM = least square mean; MCS = SF-36 Mental Component Summary; PBO = placebo; PCS = SF-36 Physical Component Summary; PGRS = Patient’s Global Rating of Severity; SE = standard error; SF-36 = Medical Outcomes Study 36-Item Short Form Health Survey Version 2 Standard. A and B: *P <.1, **P <.01, ^‡^*P* <.001 vs PBO. C and D: *P <.05, **P <.001 vs PBO. E: *P <.05, **P <.01, ***P <.001 vs PBO. F: *P <.05; **P <.01 vs PBO.

At W4, patients who received 600 mg or 1000 mg but not 200 mg of mirikizumab had a greater change from baseline in IBDQ scores compared to PBO. At 12 weeks, all mirikizumab groups had greater change from baseline in IBDQ scores compared to placebo (Figure 2A). Similarly, at week 12, IBDQ response was achieved in 58.1%, 68.8%, and 68.8% of patients in the mirikizumab 200, 600, and 1000 mg groups, respectively, compared to 45.3% in the PBO group (600 mg *P* <.1, 1000 mg *P* <.01) (Figure 2B). At W12, the change from baseline IBDQ domain scores were significantly improved at all mirikizumab dosages compared to placebo for bowel symptoms (*P* <.001), systemic symptoms (*P* <.001), emotional function (200 mg and 600 mg: *P* <.001; 1000 mg: *P* = .002), and social function (200 mg and 600 mg: *P* = .002; 1000 mg: *P* <.001) (data not shown).

Significant improvement in SF-36 Mental Component Summary (MCS) was observed at W4 with 600 mg mirikizumab (*P* = .046) and at W12 for all doses (200 mg: *P* = .008; 600 mg: *P* = .033; 1000 mg: *P* = .021), whereas significant improvements in SF-36 Physical Component Summary (PCS) were observed only at W12 with the 600 and 1000 mg doses (600 mg: *P* <.001; 1000 mg: *P* = .001) compared to PBO (Figure 2C and D). For SF-36 domain scores, changes from baseline rates were bodily pain (600 mg: *P* <.001; 1000 mg: *P* = .004), general health (600 mg: *P* = .035; 1000 mg: *P* <.001), mental health (200 mg: *P* = .017; 600 mg: *P* = .035; 1000 mg: *P* = .009), physical functioning (200 mg: *P* = .046; 600 mg: *P* = .027; 1000 mg: *P* = .010), role-emotional (200 mg: *P* = .006; 600 mg: *P* = .046), role-physical (600 mg: *P* = .001; 1000 mg: *P* = .004), social functioning (600 mg: *P* = .017; 1000 mg: *P* = .032), and vitality (200 mg: *P* = .003; 600 mg: *P* = .004; 1000 mg: *P* = .002) at particular dosing (data not shown).

Significant reduction in PGRS compared to PBO was observed as early as W4 in patients treated with 600 mg of mirikizumab (*P* = .039) and in all mirikizumab-treated patients by W12: 200 mg (*P* = .007), 600 mg (*P* <.001), and 1000 mg (*P* = .005) (Figure 2E).

Significant reduction in AP NRS was reported by patients treated with 600 mg mirikizumab compared to patients treated with placebo at W8 (*P* = .02) and in all mirikizumab treatment groups: 200 mg (*P* = .043), 600 mg (*P* = .006), and 1000 mg (*P* = .019) mirikizumab versus PBO at W12 (Figure 2F).

### QoL and PRO outcomes: maintenance period (W12-52)

**Figure 3:**
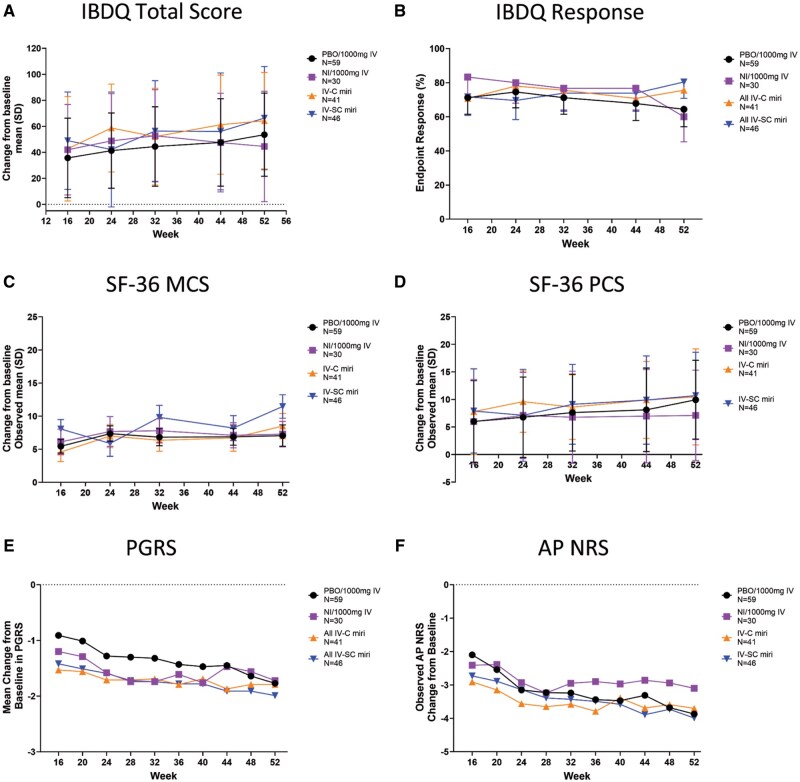
QoL and PRO assessments in the maintenance period (week 12-52) including change from baseline in IBDQ total score (A), IBDQ response (B), change from baseline in SF-36 MCS (C) and PCS (D), change from baseline in PGRS (E), and change from baseline in AP NRS (F). Abbreviations: AP NRS = Abdominal pain Numeric Rating Scale; IBDQ = Inflammatory Bowel Disease Questionnaire; IV-C = IV-Combined (200 mg, 600 mg, 1000 mg miri combined); IV-SC= IV-subcutaneous; MCS = SF-36 Mental Component Summary; NI = non-improvers; PBO = placebo; PCS = SF-36 Physical Component Summary; PGRS = Patient’s Global Rating of Severity; SD = standard deviation; SF-36 = Medical Outcomes Study 36-Item Short Form Health Survey Version 2 Standard.

There were no significant differences in mean change from baseline (from W16) at W52 in IBDQ total score (Figure 3A). The IBDQ response rates at week 52 were 75.6% (31/41), 80.4% (37/46), 64.4% (38/59), and 60.0% (18/30) in the IV-C (patients re-randomized to continue receiving IV mirikizumab treatment pooled), IV-SC, PBO/1000 mg IV, and NI/1000 mg IV groups, respectively (Figure 3B). From W12-52, treatment with mirikizumab in all groups resulted in increased IBDQ domain score rates (Table 2).

**Table 2:** QoL and PRO outcome measures in the induction, maintenance, and extension periods.

	Induction period (Wk 12), LSM (SE)				Maintenance period (Wk 52), observed mean (SD)				Extension period (Wk 104), observed mean (SD)			
	PBO IV *N *= 64	MIRI 200 mg IV *N *= 31	MIRI 600 mg IV *N *= 32	MIRI 1000 mg IV *N *= 64	PBO/1000 mg IV *N *= 59	NI/1000 mg IV *N *= 30	ALL MIRI IV-C *N *= 41	ALL MIRI IV-SC *N *= 46	PBO/1000 mg IV/300 mg SC *N *= 40	NI/1000 mg IV/300 mg SC *N *= 23	ALL MIRI IV-C/300 mg SC *N *= 33	ALL MIRI IV-SC/300 mg SC *N *= 41
**SF-PCS, CFBL**	3.1 (0.8)	4.7 (1.1)	8.0 (1.1)	6.7 (0.8)	9.9 (7.2)	7.1 (8.2)	10.5 (8.7)	10.7 (7.9)	11.3 (9.0)	9.4 (10.0)	11.7 (6.6)	10.0 (8.2)
**SF-MCS, CFBL**	2.3 (1.1)	7.5 (1.6)	6.5 (1.6)	6.1 (1.2)	7.1 (10.6)	7.3 (9.8)	8.5 (11.3)	11.5 (11.4)	7.5 (10.2)	11.5 (9.6)	9.3 (12.4)	10.4 (11.6)
**SF-36 domain scores, CFBL**												
** Physical functioning**	5.3 (2.0)	12.2 (2.9)	13.2 (2.9)	12.7 (2.1)	17.6 (16.9)	9.8 (20.6)	17.3 (18.5)	22.9 (22.3)	18.2 (21.4)	14.8 (21.7)	19.1 (17.2)	22.4 (22.8)
** Role-physical**	8.3 (2.6)	14.3 (3.6)	22.7 (3.6)	18.8 (2.6)	27.2 (23.9)	23.3 (26.6)	30.9 (25.0)	32.0 (28.2)	32.6 (26.7)	34.7 (28.4)	32.3 (20.9)	26.6 (29.3)
** Role-emotional**	2.2 (2.7)	14.8 (3.8)	11.4 (3.7)	9.4 (2.7)	14.3 (23.6)	17.3 (21.3)	13.1 (25.6)	22.4 (21.4)	14.8 (22.1)	25.4 (23.0)	17.0 (26.1)	20.0 (22.4)
** Bodily pain**	9.3 (2.6)	18.1 (3.7)	26.0 (3.7)	19.9 (2.7)	30.0 (26.7)	26.1 (27.1)	27.2 (29.0)	34.6 (25.3)	32.1 (25.7)	34.3 (30.1)	35.3 (23.9)	31.0 (27.9)
** Vitality**	6.9 (2.5)	19.6 (3.5)	19.5 (3.5)	18.0 (2.5)	22.8 (23.0)	20.2 (24.3)	28.0 (27.4)	30.8 (26.2)	25.0 (24.2)	25.6 (22.8)	26.7 (29.4)	29.1 (26.2)
** Social functioning**	11.4 (3.0)	14.0 (4.3)	23.7 (4.2)	20.4 (3.0)	23.6 (24.0)	19.7 (27.9)	29.3 (29.9)	31.1 (24.7)	25.7 (25.9)	32.5 (27.3)	31.9 (24.9)	26.0 (30.3)
** Mental health**	4.4 (2.1)	13.1 (3.0)	12.2 (3.0)	12.2 (2.1)	13.5 (19.1)	11.0 (20.3)	15.6 (20.4)	21.8 (23.6)	14.6 (18.8)	19.0 (20.6)	17.8 (23.6)	20.8 (22.7)
** General health**	5.8 (1.9)	9.4 (2.6)	12.9 (2.7)	14.6 (1.9)	19.1 (17.1)	14.0 (18.8)	23.9 (20.4)	21.0 (18.3)	22.6 (19.9)	18.9 (23.4)	26.3 (21.7)	22.6 (18.4)
** IBDQ response, *n* (%)**	29 (45.3)	18 (58.1)	22 (68.8)	44 (68.8)	38 (64.4)	18 (60.0)	31 (75.6)	37 (80.4)	32 (80.0)	18 (78.3)	27 (81.8)	32 (78.0)
** IBDQ score, CFBL**	17.1 (3.7)	41.2 (5.3)	46.6 (5.2)	42.4 (3.8)	53.6 (32.0)	44.5 (42.4)	64.3 (37.1)	66.4 (32.7)	57.2 (36.7)	62.6 (42.8)	67.1 (37.4)	57.9 (43.4)
**IBDQ domain scores, CFBL**												
** Bowel symptoms**	5.8 (1.2)	12.6 (1.7)	16.1 (1.6)	15.3 (1.2)	18.1 (11.3)	15.6 (12.3)	22.0 (12.6)	21.9 (11.9)	18.8 (11.8)	20.8 (13.5)	23.5 (11.7)	18.8 (13.2)
** Systemic symptoms**	2.8 (0.7)	7.1 (1.0)	8.3 (1.0)	6.8 (0.7)	8.8 (6.1)	7.8 (7.9)	10.5 (7.7)	10.6 (6.6)	9.4 (6.0)	10.4 (7.7)	10.1 (7.6)	9.3 (7.7)
** Emotional function**	5.7 (1.6)	15.0 (2.3)	15.1 (2.3)	12.7 (1.6)	17.7 (12.8)	13.9 (17.9)	20.6 (14.2)	22.9 (16.4)	18.9 (15.2)	20.8 (17.3)	21.8 (15.9)	20.2 (17.5)
** Social function**	2.8 (0.7)	6.9 (1.1)	6.9 (1.0)	7.1 (0.8)	8.9 (6.2)	7.3 (7.5)	11.2 (6.9)	11.1 (7.8)	10.2 (7.6)	10.7 (8.1)	11.7 (6.3)	9.5 (7.9)
** AP NRS, CFBL**	−1.0 (0.3)	−2.0 (0.4)	−2.3 (0.4)	−1.9 (0.3)	−3.9 (2.2)	−3.1 (2.4)	−3.7 (2.5)	−4.0 (2.4)	−4.2 (2.1)	−3.5 (2.6)	−4.6 (2.2)	−4.2 (2.3)
** PGRS, CFBL**	−0.4 (0.1)	−1.1 (0.2)	−1.3 (0.2)	−1.0 (0.1)	−1.8 (1.3)	−1.7 (1.1)	−1.8 (1.3)	−2.0 (1.3)	−1.9 (1.4)	−1.9 (1.4)	−2.1 (1.3)	−2.1 (1.2)

Abbreviations: AP NRS = Abdominal Pain Numeric Rating Scale; CFBL = change from baseline; IBDQ = Inflammatory Bowel Disease Questionnaire; IV = intravenous; LSM = least square means; miri = mirikizumab; NI = non-improver; PBO = placebo; PCS = Physical Component Summary; PGRS = Patient’s Global Rating of Severity; SC = subcutaneous; SD = standard deviation; SE = standard error; SF-36 = Medical Outcomes Study 36-Item Short Form Health Survey Version 2 Standard.

Although statistical comparisons were not made between treatment groups during maintenance, exploratory analysis of mean changes in SF-36 MCS (W52: IV-C: 8.5; IV-SC: 11.5; PBO/1000 mg IV: 7.1; NI/1000 mg IV: 7.3) and SF-36 PCS (W52: IV-C: 10.5; IV-SC: 10.7; PBO/1000 mg IV: 9.9; NI/1000 mg IV: 7.1) suggest that improvements during induction were sustained or further numerically increased with mirikizumab maintenance treatment, even in induction of the NI/1000 mg IV group (Figure 3C and D). All groups that received mirikizumab treatment-maintained SF-36 change from baseline domain score improvement from W12 to W52 (Table 2).

Continued numeric reductions in PGRS were observed at W52 in all mirikizumab dosing groups (IV-C: −1.9; IV-SC: −2.0; PBO/1000 mg IV: −1.8; NI/1000 mg IV: −1.7) (Figure 3E). Patients treated with PBO/1000 mg IV reported similar reductions in PGRS at W52 to patients treated with mirikizumab from W0-52 (Table 2).

Patients who received mirikizumab during induction showed improvements in AP NRS at W52 (Figure 3F). Patients treated with PBO/1000 mg IV during maintenance reported similar improvements to patients who received mirikizumab from W0-52 (W52: IV-C: −3.8; IV-SC: −4.0; PBO/1000 mg IV: −3.9; NI/1000 mg IV: −3.1) (Table 2).

### QoL and PRO outcomes: extension period (W52-104)

**Figure 4:**
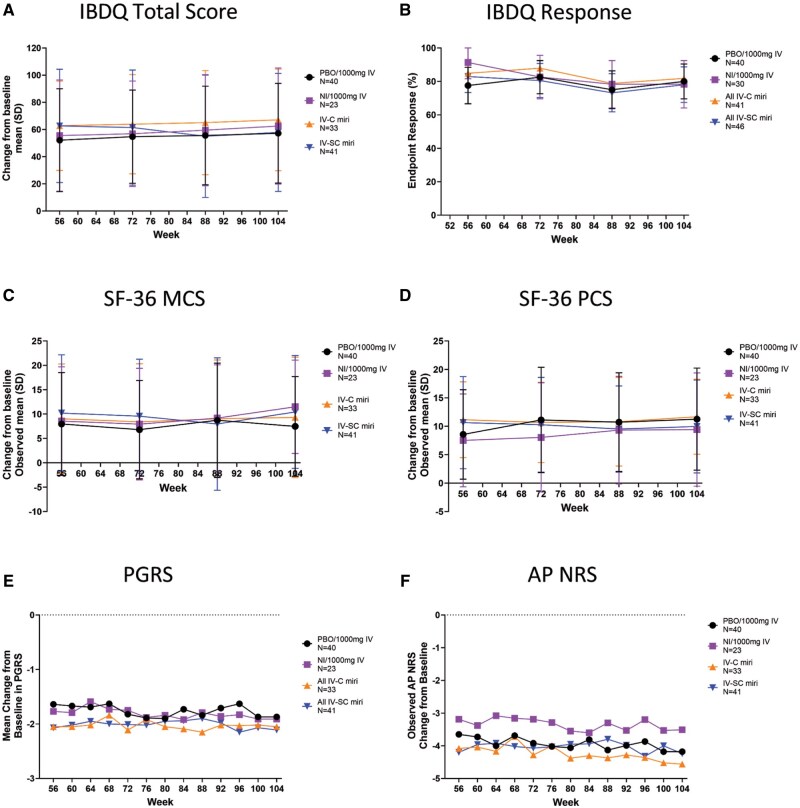
QoL and PRO assessments in the extension period (week 52-104) including change from baseline in IBDQ total score (A), IBDQ response (B), change from baseline in SF-36 MCS (C) and PCS (D), change from baseline in PGRS (E), and change from baseline in AP NRS (F). Abbreviations: AP NRS = Abdominal pain Numeric Rating Scale; IBDQ = Inflammatory Bowel Disease Questionnaire; IV-C = IV-Combined (200 mg, 600 mg, 1000 mg miri combined); IV-SC= IV-subcutaneous; MCS= SF-36 Mental Component Summary; NI = non-improvers; PBO = placebo; PCS= SF-36 Physical Component Summary; PGRS = Patient’s Global Rating of Severity; SD = standard deviation; SF-36 = Medical Outcomes Study 36-Item Short Form Health Survey Version 2 Standard.

Patients maintained efficacy, and there were no significant differences in the mean change from baseline (from W16) IBDQ total scores at W104 after 52 weeks of treatment with 300 mg SC mirikizumab (Figure 4A). At W104, the IBDQ response rates were 81.8% (27/33), 78.0% (32/41), 80% (32/40), and 78.3% (18/23) in the IV-C, SC, PBO/1000 mg IV, and NI/1000 mg IV groups, respectively (Figure 4B). From W52-104, treatment with mirikizumab in all groups resulted in increased IBDQ domain score rates (Table 2).

Statistical comparisons were not made during the extension period, but the improvements in mean changes from baseline in SF-MCS (W104: IV-C: 9.3; IV-SC: 10.4; PBO/1000 mg IV: 7.5; NI/1000 mg IV: 11.5) and SF-PCS (W104: IV-C: 11.7; IV-SC: 9.9; PBO/1000 mg IV: 11.3; NI/1000 mg IV: 9.4) appeared sustained or further numerically increased with mirikizumab extension treatment by W104 (Figure 4C and D). All groups that received mirikizumab treatment-maintained SF-36 change from baseline domain score improvement from W52 to W104 (Table 2).

PGRS mean score sustained the reduction observed in the maintenance period up to W104 (IV-C: −2.2; IV-SC: −2.1; PBO/1000 mg IV: −1.9; NI/1000 mg IV: −1.9) (Figure 4E). Patients treated with PBO/1000 mg IV reported similar reductions in PGRS at W104 to patients treated with mirikizumab W0-104 (Table 2).

All patients continued to show evidence of improvements with numerical reduction of mean AP NRS scores up to W104 (Figure 4F). Patients who received mirikizumab during induction through maintenance had similar results compared to patients treated with PBO/1000 mg IV during maintenance and extension periods (W104: IV-C: −4.7; IV-SC: −4.2; PBO/1000 mg IV: −4.2; NI/1000 mg IV: −3.5) (Table 2).

## Discussion

This study evaluated the effect of mirikizumab on QoL and PROs in patients with moderately to severely active CD through 104 weeks, showing that treatment with mirikizumab improves QoL and PROs as early as W12. After W12, IBDQ, SF-35, PGRS, and AP NRS scores appeared similar or numerically improved up to W104 suggesting that improvements were sustained. Meta-analyses on IBD^27^^,^^29^^,^^30^ have shown that generic QoL measures may underestimate the severity of IBD on patients. This suggests that disease-specific QoL measures such as IBDQ are more appropriate for studies regarding IBD. Similar to other studies,^31^^,^^32^ evaluating treatments for CD, a significant portion of patients in the SERENITY study were able to achieve IBDQ remission indicating a substantial improvement in QoL.

As early as W4, patients treated with 600 mg mirikizumab had a significantly improved IBDQ total score, PGRS, and SF-36 MCS compared to patients treated with PBO (Figure 2). By W8, patients treated with 600 mg mirikizumab had significant improvements in AP NRS compared to PBO, and by W12, all QoL scores were significantly improved compared to PBO. Interestingly, the 600 mg dose of mirikizumab significantly improved all QoL and PRO outcomes measured earlier than 1000 mg, consistent with an earlier publication.^22^ However, change from baseline in fatigue measured by FACIT-Fatigue showed no significant improvement until W12 in all doses.^33^ The 600 mg dose was then prioritized to move forward into phase 3 testing accordingly.

Our results indicate that treatment with mirikizumab improves IBDQ scores, which is a common measure of QoL in adults with CD, but other important PROs such as abdominal pain.^30^ Additionally, up to 60% of patients with CD experience abdominal pain, which impacts daily life and can result in increases psychosocial burdens for patients.^27^ Patients that received mirikizumab reported significant improvements in abdominal pain, further highlighting the consistency of the benefit demonstrated overall. Collectively, this highlights the impact that mirikizumab has on patients with IBD by disclosing the improvements in QoL and an important PRO.

In another study focusing on ulcerative colitis, improvements measured by IBDQ and SF-36 were observed in patients treated with mirikizumab compared to placebo.^24^ This is consistent with the improvements observed in this study, where from W52 to W104, treatment with mirikizumab in all groups resulted in increased IBDQ domain score rates and maintained SF-36 change from baseline domain score improvements from W52 to W104 (Table 2). Together, these show that mirikizumab treatment improves IBDQ and SF-36 in IBD.

This study was limited as the data were from a phase 2 study with small patient numbers based on a predominately White patient population, which may reduce the generalizability of the results. It is important to also indicate that patients in clinical trials are followed more closely compared to standard clinical practice, which can impact QoL outcomes. Also, there was a lack of a placebo control during the maintenance and extension phases where if the PBO/1000 mg IV mirikizumab-treated patients were used as a comparator, this could have resulted in an underestimation of the improvements observed. After W12, statistics are exploratory due to low numbers and the lack of a placebo control and cannot be directly compared to the results up to W12. Other factors related to QoL were not included in this study, such as work productivity, although this was investigated in the VIVID-1 phase 3 study.^34^ Other limitations of this study are those inherent to post hoc analyses of clinical study data. These findings have been validated in the VIVID-1 phase 3 study (NCT03926130).^34^^,^^35^

## Conclusion

Treatment with mirikizumab improved QoL and PRO measures by 12 weeks in patients with moderate-to-severely active CD. These results were sustained at 52 weeks with both IV and SC treatment and sustained up to 104 weeks with 52 weeks of SC treatment. These QoL results, taken with the existing and emerging clinical and endoscopic efficacy and safety data, support a role for mirikizumab in treating moderately-to-severely active CD.

## Supplementary Material

otag018_Supplementary_Data

## Data Availability

Lilly provides access to all individual participant data collected during the trial, after anonymization, with the exception of pharmacokinetic or genetic data. Data are available to request 6 months after the indication studied has been approved in the US and EU and after primary publication acceptance, whichever is later. No expiration date of data requests is currently set once data is made available. Access is provided after a proposal has been approved by an independent review committee identified for this purpose and after receipt of a signed data sharing agreement. Data and documents, including the study protocol, statistical analysis plan, clinical study report, and blank or annotated case report forms, will be provided in a secure data sharing environment. For details on submitting a request, see the instructions provided at www.vivli.org.
