# Supplementary material for: Impact of mirikizumab on patient-reported outcomes and quality of life in patients with Crohn’s disease: results from the phase 2 SERENITY study
Source: Crohns Colitis 360. 2026 Mar 11;8(1):otag018. doi: 10.1093/crocol/otag018 (PMC13017000; doi:10.1093/crocol/otag018)
Supplement: otag018_Supplementary_Data [file otag018_supplementary_data.docx]

**Supplemental Methods**

**Inclusion Criteria**

Subjects will be eligible for the study only if they meet all of the following criteria within the

screening period, which is ≤28 days prior to the start of study treatment, unless specifically

defined:

*Type of Patient and Disease Characteristics*

[1] have had a diagnosis of Crohn’s disease for ≥3 months before baseline

[2] have active Crohn’s disease as defined absolute stool frequency (SF) ≥4 (loose and watery stools defined as Bristol Stool Scale Category 6 or 7) AND/OR abdominal pain (AP) ≥2 at baseline (refer to Section 9.1.2 for details)

[3] have an Simple Endoscopic Score for Crohn’s Disease (SES-CD) score ≥7 (centrally read) for subjects with ileal-colonic or ≥4 for subjects with isolated ileal disease within 14 days before the first dose of study treatment

*Prior Inflammatory Bowel Disease Treatment*

[4] must have received prior treatment for Crohn’s disease (according to either “a)” or “b)” below or combination of both):

a) history of inadequate response to, or failure to tolerate, treatment with aminosalicylates, 6-mercaptopurine (6-MP) or azathioprine (AZA), or oral or IV corticosteroids or history of corticosteroid dependence (an inability to successfully taper corticosteroids without return of Crohn’s disease)

OR

b) have received treatment with ≥1 biologic agents (such as tumor necrosis factor [TNF] antagonists, vedolizumab, experimental biologic Crohn’s disease therapeutics) with or without documented history of failure to respond to or tolerate such treatment. The treatment must have been discontinued according to the following timeline:

- anti-TNF therapy at least 8 weeks before baseline
- vedolizumab treatment at least 12 weeks before baseline
- experimental biologic Crohn’s disease therapy at least 8 weeks before baseline.

[5] may be receiving a therapeutic dosage of the following drugs:

- Oral 5-aminosalicylic (ASA) compounds: if the prescribed dose has been stable for at least 3 weeks before screening colonoscopy or stopped treatment at least 3 weeks prior to screening colonoscopy.
- Oral corticosteroids must be at a prednisone-equivalent dose of ≤20 mg/day, or ≤9 mg/day of budesonide, and have been at a stable dose for at least 3 weeks prior to the screening colonoscopy. If stopping oral corticosteroid treatment prior to baseline, they must be stopped at least 3 weeks prior to screening colonoscopy.
- AZA, 6-MP, or methotrexate (MTX): if the prescribed dose has been stable for at least 4 weeks before screening endoscopy. Subjects who have discontinued therapy with AZA, 6-MP, or MTX must have stopped the medication at least 4 weeks prior to screening endoscopy to be considered eligible for enrollment.
- Crohn’s disease-specific antibiotics: if the prescribed dose has been stable 4 weeks prior to baseline or stopped treatment at least 3 weeks prior to screening endoscopy.

*Subject Characteristics*

[6] Male subjects agree to use a reliable method of birth control during the study and for 3 months, or which is greater than 5 half-lives, after the last dose of investigational product.

[7] Women of childbearing potential must agree to either remain abstinent or use effective methods of contraception for the entirety of the study. Abstinence or contraception must continue 3 months following completion of study drug administration, which is greater than 5 half-lives:

- Women of childbearing potential must test negative for pregnancy prior to initiation of treatment as indicated by a negative serum pregnancy test at the screening visit followed by a negative urine pregnancy test within 24 hours prior to exposure.
- Two effective methods of contraception will be used. The subject may choose to use a double barrier method of contraception. Barrier protection methods without concomitant use of a spermicide are not a reliable or acceptable method. Thus, each barrier method must include use of a spermicide (that is, condom with spermicide, diaphragm with spermicide, female condom with spermicide). It should be noted that the use of male and female condoms as a double barrier method is not considered acceptable due to the high failure rate when these methods are combined.

[8] Women not of childbearing potential may participate and include those who are:

- infertile due to surgical sterilization (hysterectomy, bilateral oophorectomy, or tubal ligation), congenital anomaly such as Müllerian agenesis; or
- post-menopausal – defined as one of the following:
  - a woman at least 50 years of age with an intact uterus, not on hormone therapy, who has had either
    - cessation of menses for at least 1 year, or
    - at least 6 months of spontaneous amenorrhea with a follicle-stimulating hormone (FSH) >40 mIU/mL
  - a woman ≥55 years of age not on hormone therapy, who has had at least 6 months of spontaneous amenorrhea
  - a woman ≥55 years of age with a diagnosis of menopause prior to starting hormone replacement therapy

[9] venous access sufficient to allow blood sampling and IV administration as per the protocol

[10] are willing and able to complete the scheduled study assessments, including endoscopy

[11] have an adequate organ function, including:

- hematologic: absolute neutrophil count ≥1.5 x 109/L (≥1.5 x 103/μL or ≥1.5 GI/L), platelet count ≥100 x 109/L (≥100 x 103/μL or ≥100 GI/L), hemoglobin level ≥10.0 g/dL (≥100 g/L), absolute lymphocyte count >500 cells/μL (>0.50 x 103/μL or >0.50 GI/L), and total white blood cell count ≥3.0 x 109/L (≥3.0 x 103/μL or ≥3.0 GI/L)
- chemistry: serum creatinine, total bilirubin level (TBL; subjects with Gilbert’s syndrome must have serum direct bilirubin <1.5 mg/dL), alkaline phosphatase (ALP), alanine aminotransferase (ALT), and aspartate aminotransferase (AST) levels less than or equal to 2 times the upper limit of normal (≤2X ULN).

[12] have given written informed consent approved by the ethical review board (ERB) governing the site

[13] are male or female subjects ≥18 and ≤75 years of age at the time of initial screening.

**Exclusion Criteria**

Subjects will be excluded from study enrollment if they meet any of the following criteria within the screening period, which is ≤28 days prior to the start of study treatment, unless specifically defined:

*Study Disease Conditions or Treatments*

[14] have complications of Crohn’s disease such as strictures, stenoses, or any other manifestation for which surgery might be indicated or could confound the evaluation of efficacy

[15] diagnosis of conditions affecting the digestive tract, such as ulcerative colitis (UC), indeterminate colitis, fistulizing disease, abdominal or perianal abscess, adenomatous colonic polyps not excised, colonic mucosal dysplasia, and short bowel syndrome

[16] have had any kind of bowel resection, diversion, or placement of a stoma within 6 months or any other intra-abdominal surgery within 3 months prior to screening

[17] have received any of the following for treatment of Crohn’s disease:

- 6-thioguanine (6-TG), cyclosporine, tacrolimus, sirolimus, pentoxifylline, or mycophenolate mofetil within 8 weeks prior to baseline
- corticosteroid enemas, IV corticosteroids, corticosteroid suppositories, or topical treatment within 3 weeks prior to screening colonoscopy
- rectal 5-ASA within 3 weeks prior to screening colonoscopy
- have used apheresis (for example, Adacolumn apheresis) ≤2 weeks prior to screening.

[18] have previous exposure to any biologic therapy targeting IL-23p19 either licensed or investigational. Patients who have been exposed to ustekinumab are excluded from the study, except for patients who have received a single IV “induction” dose of ustekinumab, at a marketed dose at least 12 weeks prior to the baseline, and discontinued treatment prior to receiving a subcutaneous (SC) “maintenance” dose, for reasons other than inadequate response, loss of response, or intolerance to medication.

[19] have received natalizumab or agents that deplete B or T cells (for example, rituximab, alemtuzumab, or visilizumab) within 12 months of screening, or if after receiving these agents, evidence is available at screening of persistent depletion of the targeted lymphocyte population

[20] have been treated with any investigational drug for Crohn’s disease within 8 weeks prior to baseline or 5 half-lives of the drug (whichever is longer) or with interferon therapy within 8 weeks before baseline

*General Eligibility Criteria*

[21] have evidence of active or latent tuberculosis (TB) (see full TB testing details below)

[22] have had any malignancy within 5 years of screening, except for basal cell or squamous epithelial carcinoma of the skin that has been resected with no evidence of metastatic disease for at least 3 years or cervical carcinoma in situ with no evidence of recurrence within 5 years of screening

[23] have an abnormality in the 12-lead electrocardiogram (ECG) that, in the opinion of the investigator, increases the risks associated with participating in the study

[24] increases the risks associated with participating in the study if the presence or history within 12 months prior to screening of significant uncontrolled cerebrocardiovascular (for example, myocardial infarction, unstable angina, unstable arterial hypertension, moderate-to-severe heart failure [New York Heart Association class III/IV], or cerebrovascular accident) and presence of respiratory, hepatic, renal, gastrointestinal, endocrine, hematologic, or abnormal laboratory values at screening that, in the opinion of the investigator, pose an unacceptable risk to the subject if participating in the study or of interfering with the interpretation of data

[25] presence of significant uncontrolled neuropsychiatric disorder, have history of a suicide attempt or have a score of 3 on Item 12 (Thoughts of Death or Suicide) of the Quick Inventory of Depressive Symptomatology–Self Report (16 Items) (QIDS-SR16) at screening (Visit 1) or baseline (Week 0; Visit 2)

[26] are investigator site personnel directly affiliated with this study and/or their immediate families. Immediate family is defined as a spouse, parent, child, or sibling, whether biological or legally adopted.

[27] are Lilly employees or employees of third party organizations (TPOs) involved with the study

[28] are currently enrolled in a clinical trial involving an investigational product or non-approved use of a drug or device or are concurrently enrolled in any other type of medical research not scientifically or medically compatible with this study, per investigator judgment

[29] have previously completed or withdrawn from this study or any other study investigating LY3074828. This criterion does not apply to subjects undergoing rescreening procedures.

[30] have received live, attenuated vaccine(s) within 2 months of screening or intend to receive such during the study; vaccines should be avoided for 2 months after the last dose of study drug. Uses of nonlive (inactivated) vaccinations are allowed for all subjects.

[31] have human immunodeficiency virus/acquired immune deficiency syndrome (HIV/AIDS) or test positive for antibodies at screening

[32] have hepatitis B or test positive for hepatitis B virus (HBV) at screening, defined as (1) positive for hepatitis B surface antigen or (2) positive for anti-hepatitis B core antibody (HBcAb+) and positive confirmatory polymerase chain reaction (PCR) for HBV, regardless of anti-hepatitis B surface antibody status

[33] have hepatitis C or test positive hepatitis C virus at screening, defined as positive result for hepatitis C antibody and positive confirmatory PCR test for hepatitis C virus

[34] had *Clostridium difficile* (*C diff*) infection within 60 days of screening or test positive at screening or other intestinal pathogen with 30 days before screening endoscopy. Subject must not have signs of an ongoing infection related to an intestinal pathogen.

[35] have any clinically significant extra-intestinal infection or opportunistic, chronic, or recurring infection within 6 months before screening. Examples include but are not limited to infections requiring IV antibiotics, hospitalization, or prolonged treatment.

[36] have received a systemic (including oral) anti-infective agent for an infection within 28 days of baseline

[37] are pregnant, lactating, or planning pregnancy (both men and women) while enrolled in the study or within 3 months after receiving the last dose of study agent

[38] have significant allergies to humanized monoclonal antibodies or any components of the LY3074828 product formulation

[39] history of alcohol or other drug abuse within the last year

[40] are unsuitable for inclusion in the study in the opinion of the investigator or sponsor for any reason that may compromise the subject’s safety or confound data interpretation.

**TB testing details**

Posterior-anterior view chest radiography (CXR) will be obtained at screening (unless local standards dictate posterior-anterior and lateral views), unless the radiographs or medical report from chest radiography performed within 3 months before initial screening (per local standard of care for TB evaluation) is available to the investigator for review. In addition, subjects will be tested for evidence of active or latent TB. A positive TB test result is indicated by a purified protein derivative (PPD) skin test response ≥5 mm induration documented 48 to 72 hours after test application (regardless of Bacillus Calmette-Guerin vaccination history). In countries where the QuantiFERON-TB Gold test (or equivalent, for example, T-SPOT) is available and is preferred (in the judgment of the investigator) as an alternative to the PPD skin test for the evaluation of TB infection in a subject, that test may be used instead of the PPD test. Retesting following a positive test is allowed in patients, who in the opinion of the investigator, are unlikely to be infected with *Mycobacterium tuberculosis*. In this circumstance, 2 positive tests is considered evidence of active or latent TB infection. Patients in whom retesting has been performed must be discussed with the medical monitor prior to inclusion in the study. If the QuantiFERON-TB Gold test is indeterminate, 1 retest is allowed. If the retest is indeterminate, then the subject is excluded from the study.

Subjects with documentation of negative TB test results within 3 months before initial screening may not need to repeat TB testing at screening based on judgment of the investigator. Documentation of this previous test result must include a record of the size (in millimeters) of the induration response. A PPD test recorded as “negative” without documenting the size of induration (in millimeters) will not be acceptable and will require a retest. However, subjects with a PPD skin test response ≥5 mm induration or a positive QuantiFERON-TB Gold test result at screening and no other evidence of active TB may be rescreened once and enrolled according to the following requirements:

- after receiving at least 4 weeks of appropriate ongoing prophylactic therapy for latent TB as per local standard of care
- no evidence of treatment hepatotoxicity (ALT and AST levels must remain ≤2xULN) upon retesting of serum ALT and AST levels before randomization)

Such subjects must continue and complete appropriate latent TB therapy during the course of the study to remain eligible and must continue to meet all other inclusion and exclusion criteria for participation. Subjects who have a documented history of completing an appropriate TB prophylaxis regimen with no history of re-exposure since their treatments were completed and no evidence of active TB are eligible to participate in the study; these subjects should not undergo PPD testing. Subjects who have had household contact with a person with active TB must be excluded unless appropriate and documented prophylaxis for TB has been given, as described above. Subjects with any history of active TB are excluded from the study, regardless of previous or current TB treatments.

**Supplemental Figures**

**Supplemental Figure 1: CONSORT diagram**

**
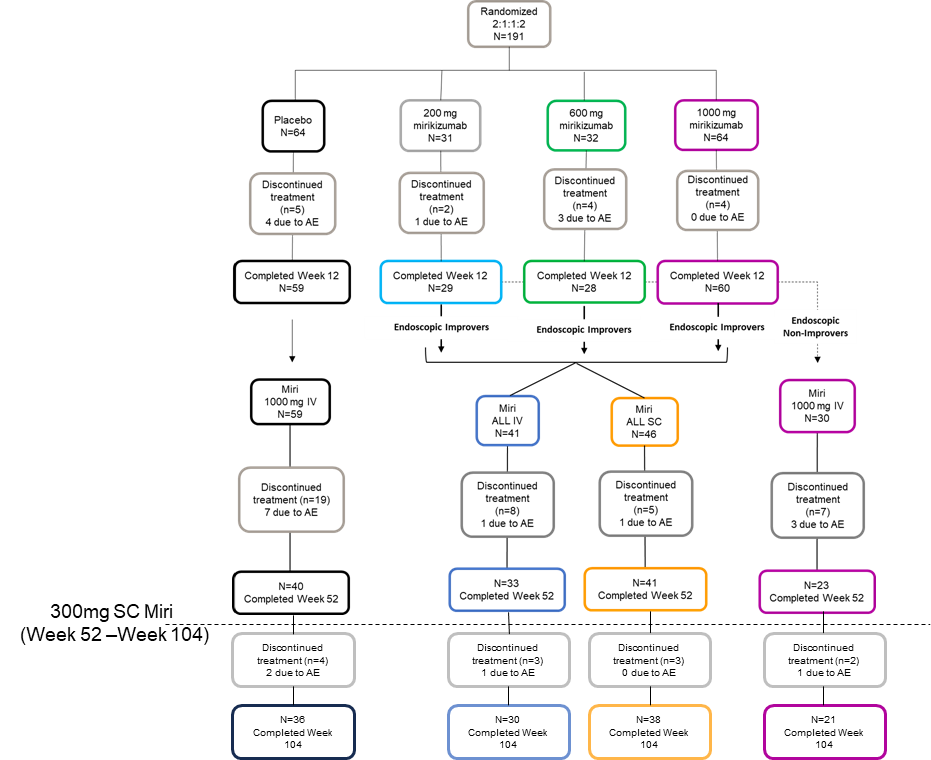
**
